# Heath-related quality of life in thyroid cancer patients following radioiodine ablation

**DOI:** 10.1186/1477-7525-9-33

**Published:** 2011-05-13

**Authors:** David Taïeb, Karine Baumstarck-Barrau, Frédéric Sebag, Cécile Fortanier, Catherine De Micco, Anderson Loundou, Pascal Auquier, Fausto F Palazzo, Jean-françois Henry, Olivier Mundler

**Affiliations:** 1 Service central de Biophysique et de Médecine Nucléaire, centre hospitalo-universitaire de la Timone, 264 rue Saint-Pierre 13385 Marseille Cedex 5, France; 2 Unité d'Aide Méthodologique à la Recherche Clinique et Épidémiologique. Faculté de Médecine, 27 Bd Jean Moulin, 13385 Marseille Cedex 5, France; 3 Service de Chirurgie Générale et Endocrinienne, centre hospitalo-universitaire de la Timone, 264 rue Saint-Pierre 13385 Marseille Cedex 5, France; 4 Faculté de Médecine, Institut National de la Santé et de la Recherche Médicale (U555), 13385 Marseille Cedex 5, France; 5 Service de Santé Publique et de l'Information Médicale, centre hospitalo-universitaire Nord, chemin des Bourrely, 13015 Marseille, France

## Abstract

**Background:**

There is limited information about the medium to long-term health-related quality of life (QOL) in thyroid cancer patients after initial therapy and the existing studies suffer from limitations. The aim of the study was to assess the determinants of medium-term QOL after the initial therapy.

**Methods:**

Following a total thyroidectomy, 88 thyroid cancer patients received either rhTSH or hypothyroid-assisted radioiodine ablation (RRA) using 3.7 GBq (100 mCi) of radioiodine. QOL evaluation of the patients using the validated Functional Assessment of Chronic Illness & Therapy (FACIT) was performed at the time of inclusion (t0) and later at the 9-month post-RRA (t1).

**Results:**

83 patients were eligible for the final evaluation. Medium-term FACIT scores were not statistically different between t0 and t1 patients. All but one domain of the QOL score was similar between t0 and t1. Using a multivariate analysis, only age and immediate postoperative QOL scores were found to be determinants of the overall medium term 9-month QOL scores. Analysis showed that 'high QOL levels' (baseline and 9-month) and 'no depression', 'low anxiety levels', were associated with '<45yrs', 'men', 'partner', and 'rhTSH stimulation'.

**Conclusions:**

The use of radioiodine ablation does not seem to affect the medium term QOL scores of patients. Medium-term QOL is mainly determined by pre-ablation QOL. The assessment of baseline QOL might be interesting to evaluate in order to adapt the treatment protocols, the preventive strategies, and medical information to patients for potentially improving their outcomes.

## Background

Most well-differentiated thyroid cancers (WDTC) are treated with a total thyroidectomy followed by selective use of radioiodine for remnant ablation (RRA) [1-6]. Survival rates are excellent but poor quality of life (QOL) outcomes have been reported in thyroid cancer patients. The use of recombinant TSH (rhTSH) for RRA improves QOL during the peri-ablation period but its impact beyond this period remains to be determined in a model including other factors that contribute to influence QOL [7,8]. There is limited information about the medium to long-term quality of life (QOL) and the existing studies suffer from limitations including a cross-sectional design [9-14], a small sample size [15,16], the small number of QOL domains assessed [10,16] and the absence of baseline QOL data.

In our previous report, we found that the 9-month QOL (medium term QOL) did not differ according to the TSH stimulation method (rhTSH or hypothyroid-assisted RAA) but we did not take into account the QOL potential confounding variables including the baseline QOL status of patients [8].

The aim of the present prospective study was to identify the determinants of the medium term QOL after complete initial therapy.

## Methods

### Study design and data collection

This is a longitudinal study. Newly diagnosed well-differentiated papillary or follicular thyroid cancer patients were included. Subjects were staged pT1-T3, N0-Nx-N1, M0 (if <5 nodes and without extracapsular spread), had total thyroidectomy and underwent RRA (either rhTSH or hypothyroid-assisted RRA, using 3.7 GBq radioiodine). The patients were assessed at the time of study inclusion (t0) and at the 9 month-post-RRA follow-up control (t1). The following data were collected: socio-demographic parameters, pTNM stage, clinical data, anxiety (Spielberger trait anxiety inventory) [17,18], depression (self-administered Beck Depression Inventory, BDI) [19], and QOL (functional assessment of chronic illness therapy, FACIT) scores [20-22]. This study was integrated in a prospective randomized study previously described in detail ^8^, in which the primary objective was to compare the impact and the efficacy of two TSH stimulation procedures. The aim of this present report was a secondary objective of the original protocol.

### Instruments to assess anxiety, depression and quality of life

1. The level of anxiety was assessed with the state scale of the Spielberger trait anxiety inventory (20 items, scale range 20-80, higher scores corresponding to higher levels of anxiety).

2. The BDI score range is 0-39, with higher scores indicating worsening depression (score 0-<4: no depression, 4-<8: mild depression, 8-<30: moderate depression; score >30: severe depression).

3. The QOL was assessed using the functional assessment of chronic illness therapy. Functional assessment of chronic illness therapy (FACIT) is a well validated and widely used tool for evaluation of QOL in cancer patients. It includes the generic CORE questionnaire - functional assessment of cancer therapy-general (FACT-G) - which contains general questions divided into four primary QOL domains (a total of 27 items): physical well-being (PWB, 7 items, 0-28), social/family well-being (SWB, 7 items, 0-28), emotional well-being (EWB, 6 items, 0-24), and functional well-being (FWB, 7 items 0-28), and an additional fatigue subscale (FS, 13 items, 0-52) directly related to the impact of fatigue on daily activities. Three scores can be derived: a FACIT-F trial outcome index (TOI) corresponding to the sum of the PWB, FWB and FS subscales (range from 0 to 108), a FACT-G total score corresponding to the sum of the first four subscales PWB, SWB, EWB, FWB (range from 0 to 108), a FACIT-F total score corresponding to the sum of the FACT-G and the FS (range from 0 to 160). Higher scores are associated with higher QOL levels.

### Statistical analysis

Data were expressed in mean and standard deviations (SD) or median and ranges depending on the parametric or non-parametric distribution of the variable. Mean comparisons of QOL scores between different sub-groups (gender, age, educational level, marital status, children, occupational status, tumour staging, 1-/2-stage thyroidectomy, initial remnant ablation, depression) were performed using Mann-Whitney tests or Student's t-tests. Associations between QOL scores and continuous variables (anxiety level, interval surgery/^131^I, baseline QOL) were analyzed using Pearson's correlation tests. Multivariate analyses using multiple linear regression (forward-stepwise selection) were performed to determine variables potentially linked to medium term QOL levels. The FACIT-F, the FACIT-G, the FACIT-F TOI, and each of the five domains were considered as separate dependent variables. The variables relevant to the models were selected from the univariate FACIT-F total score analysis, based on a threshold p-value ≤0.20 (gender, age group, marital status, depression, anxiety, and baseline QOL level). Initial remnant ablation was included as an additional variable in the models owing to its clinical interest. The final models incorporated the standardized beta coefficients. The independent variables with the higher standardized beta coefficients are those with a greater relative effect on QOL. The statistical analyses were performed using the SPSS version 15.0 software package (SPSS Inc., Chicago, IL, USA). All the tests were two-sided. The statistical significance threshold was defined as *p *< 0.05. To further explore the relation between QOL levels (baseline and 9-month QOL) and the previous selected variables (gender, age group, marital status, depression, initial RAA, anxiety), a complementary multiple correspondence analysis (MCA), allowing the detection of clusters, was conducted using SPAD 3.21.

The MCA is a factor analysis approach. MCA may be considered to be an extension of simple correspondence analysis to more than two variables. MCA is used to produce a graphical representation of a set of categorical variables, based on all possible pairs of cross tabulations. MCA was performed projecting the variables onto a succession of two-dimensional planes. The relationship between variables can be deduced from the relative positions of the modalities of the variables on the planes [23].

QOL and anxiety scores were arbitrarily categorised using their median/25-75^th ^percentile values to define four classes: 1-very low/2-low/3-high/4-very high QOL levels or anxiety. All other variables were dichotomised in two or more categories. Patient characteristics (illustrative variables) were projected on the plane in order to detect the strength of the association with 9-month QOL, baseline QOL, depression, and anxiety (active variables) [24].

## Results

### Patients characteristics

Eighty eight consecutive patients were enrolled of whom 83 patients were eligible for the final evaluation (2 patients re-operated before t1, 1 patient lost to follow-up, 2 with incomplete data). The socio-demographic, clinical features and self-reported data are detailed in Table 1. The mean age of the sample was 46.9 years (SD 14.2), and the men:women ratio was 0.17. Approximately two thirds of patients declared having a partner, had high school educational level or above, had at least one child, and were in employment. According to our inclusion criteria, most tumours were considered low-risk for persistent disease. Thyroidectomy was performed in one stage in more than 80% of cases. More than 20% of the sample suffered from moderate or severe depression at baseline. At the 9-month follow-up control, only one patient had persistent disease.

**Table 1:** Baseline characteristics of the sample (*n *= 83).

		N (%) M ± SD^**§**^ **M [IQR]**^**§§**^
Age		46.91 ± 14.20

Gender	Male	12 (14.5)
	Female	71 (85.5)

Educational level	Middle school	29 (34.9)
	High school	54 (65.1)

Marital status	Single	29 (34.9)
	Partner	54 (65.1)

Children	0	18 (21.7)
	> = 1	65 (78.3)

Occupational status	Not working	35 (42.2)
	Worker or student	48 (57.8)

Tumour T stage	T1	37 (48.1)
	T2	23 (29.9)
	T3	17 (22.1)

Tumour N stage	N0	28 (33.7)
	N1	14 (16.9)
	Nx	35 (42.2)

Thyroidectomy	One-stage	65 (82.3)
	Two-stage	14 (17.7)

Initial RAA	Hypo	41 (49.4)
	rhTSH	42 (50.6)

Interval surgery/^131^I		41 [20-45]


Depression (BDI)	No	47 (56.6)
	Mild	18 (21.7)
	Moderate	16 (19.3)
	Severe	2 (2.4)

Anxiety level*	STAI [20-80]	41.03 ± 10.53

QOL**	FACIT-F [0-160]	118.63 ± 22.79
	FACIT-G [0-108]	81.02 ± 14.13
	FACIT-F TOI [0-108]	78.71 ± 18.42
	PWB [0-28]	23.78 ± 4.14
	SWB [0-28]	21.61 ± 5.08
	EWB [0-24]	18.31 ± 3.76
	FWB [0-28]	17.31 ± 5.90
	FS [0-52]	37.61 ± 10.73

^§^M ± SD: mean ± standard deviation

^§§^M [IQR]: median [interquartile range]

*the higher the score, the higher anxiety level

**the higher the score, the higher the QOL level

PWB Physical wellbeing, SWB Social/family well-being, EWB Emotional well-being, FWB Functional well-being, FS Fatigue

FACIT-G (PWB, SWB, EWB, and FWB subscales), FACIT-F (FACT-G, and FS subscale), FACIT-F TOI (PWB, FWB, and FS subscales)

### Clinical and sociodemographic factors linked to QOL

**Figure 1:**
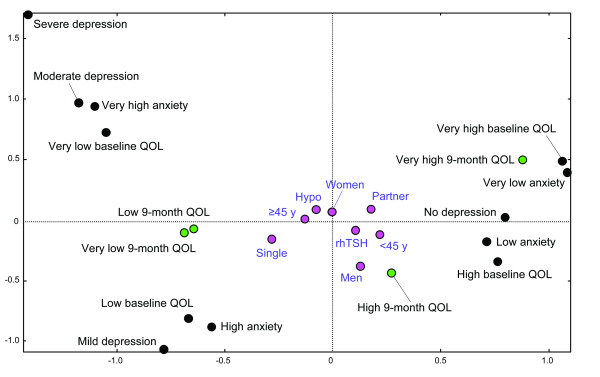
**Multiple correspondence analysis (MCA), plane of the first two factorial axes (factor 1 and factor 2) representing relationship with the 9-month QOL **Green circles: 9-month QOL (very low/low/high/very high); Black circles: anxiety (very low/low/high/very high), depression (no/mild/moderate/severe), baseline QOL (very low/low/high/very high); Pink circles: patients' parameters; single/partner, <45y/> = 45y, men/women, hypo/rhTSH.

Univariate analyses are detailed in Table 2. The 9-month FACIT-F score was statistically linked to gender, age, depression, anxiety, and baseline FACIT-F score. Older patients reported significantly worse scores for the three combined scores (FACIT-F, FACT-G, and FACIT-F TOI), and for SWB, FWB, and FS dimensions. Men had significantly better scores for two of the three scores (FACIT-F and FACIT-F TOI), and for two dimensions (PWB and FS). Depression and anxiety were always significantly related to lower QOL (except depression and the PWB dimension). All baseline QOL levels were positively correlated with 9-month QOL levels. None of the scores and domains were linked with educational level, marital status, occupational status or having children. A trend towards higher QOL levels was observed in non-working people, without children, with a partner and with a higher educational level. Means scores did not differ according to tumour staging (T or N) and thyroidectomy stage (one- or two-stage). Interval surgery/131I was also not correlated with 9-month QOL. Multivariate models are detailed in Table 3. The selected variables were gender, age group, marital status, initial RAA, depression, anxiety, and baseline QOL level. No links were found between the 9-month QOL and the modality of TSH stimulation. Marital status, baseline anxiety and depression were not linked to QOL, except SWB which was altered in subjects with initial depression. Baseline QOL directly influenced the QOL at the follow-up control. Older patients reported lower QoL levels in the 3 scores, and 2 of the 5 dimensions (FWB and FS). The PWB dimension was the single dimension influenced by gender, indicating a lower score for women. Figure 1 shows the results of the MCA regarding the relationship between 9-month QOL and other characteristics. Three clusters can be isolated in accordance with the results of the linear regression. In the right of the graph, a first cluster including 'no depression', 'low anxiety levels', and 'high QOL levels' (baseline and 9-month) presented close similarities with 'younger', 'men', 'partner', and 'rhTSH' response modalities. 'Mild depression', 'high anxiety', 'low baseline QOL' are associated with 'low' and 'very low 9-month QOL' and represent a second cluster. 'Single' seems included in this second cluster as 'partner' seems more closed of the first one, but 'age group' is probably a confounding factor as demonstrated by the linear regression. A third cluster groups together 'moderate depression', 'severe depression', 'very high anxiety level', and 'very low baseline QOL'.

**Table 2:** Associations between 9-month FACIT scores/dimensions and sociodemographics, baseline clinical characteristics

	FACIT-F	FACIT-G	FACIT F TOI	PWB	SWB	EWB	FWB	FS
	**[0-160]**	**[0-108]**	**[0-108]**	**[0-28]**	**[0-28]**	**[0-24]**	**[0-28]**	**[0-52]**

Gender*								

Men	131.64 ± 15.91	87.14 ± 11.43	90.10 ± 10.95	25.00 ± 2.59	21.81 ± 5.52	19.75 ± 2.56	20.58 ± 3.96	44.50 ± 7.76
Women	118.10 ± 27.62	80.26 ± 16.54	78.34 ± 21.55	22.51 ± 5.26	21.14 ± 5.28	18.67 ± 4.14	17.94 ± 5.67	37.98 ± 11.76
p	**0.026**	0.172	**0.008**	**0.017**	0.690	0.396	0.126	**0.070**

Age group*								

< 45 y	128.91 ± 17.70	86.29 ± 11.73	86.32 ± 14.50	23.79 ± 4.67	23.12 ± 3.58	19.34 ± 3.38	20.04 ± 4.72	42.39 ± 7.31
> = 45 y	113.86 ± 29.90	77.48 ± 17.73	75.68 ± 23.26	22.09 ± 5.34	19.88 ± 5.95	18.42 ± 4.36	17.09 ± 5.68	36.66 ± 13.16
p	**0.009**	**0.012**	**0.020**	0.164	**0.005**	0.338	**0.023**	**0.020**

Educational level*								

Middle school	116.29 ± 24.85	79.57 ± 14.59	76.92 ± 19.80	22.29 ± 5.23	20.83 ± 4.77	18.70 ± 3.43	17.74 ± 4.97	37.12 ± 11.28
High school	122.21 ± 27.36	82.20 ± 16.75	81.82 ± 21.08	23.19 ± 4.92	21.45 ± 5.57	18.90 ± 4.22	18.65 ± 5.79	39.96 ± 11.51
p	0.358	0.492	0.328	0.455	0.625	0.832	0.488	0.305

Marital status*								

Single	115.00 ± 28.40	78.70 ± 16.90	76.07 ± 22.25	22.52 ± 5.31	20.69 ± 5.04	18.24 ± 4.21	17.24 ± 5.89	36.31 ± 12.13
Partner	123.35 ± 25.10	82.82 ± 15.43	82.65 ± 19.45	23.10 ± 4.87	21.56 ± 5.44	19.18 ± 3.78	18.98 ± 5.22	40.63 ± 10.81
p	0.182	0.272	0.178	0.622	0.484	0.311	0.178	0.109

Number of children*								
0	123.50 ± 25.83	83.67 ± 17.18	82.00 ± 19.27	23.39 ± 5.28	22.56 ± 4.33	18.94 ± 4.40	18.78 ± 5.67	39.83 ± 9.18
> = 1	119.20 ± 26.87	80.61 ± 15.72	79.61 ± 21.19	22.74 ± 4.97	20.85 ± 5.51	18.80 ± 3.84	18.21 ± 5.50	38.75 ± 12.11
p	0.551	0.479	0.670	0.631	0.232	0.895	0.705	0.727

Occupational status*								

Not working	121.49 ± 24.53	81.62 ± 14.53	81.48 ± 19.41	23.19 ± 4.73	20.46 ± 5.83	19.55 ± 2.85	18.42 ± 5.14	39.87 ± 11.18
Worker or student	119.34 ± 28.01	80.93 ± 17.35	79.28 ± 21.62	22.59 ± 5.31	21.82 ± 4.98	18.24 ± 4.52	18.28 ± 5.90	38.41 ± 11.7
p	0.908	0.998	0.681	0.680	0.273	0.279	0.369	0.533

Initial remnant ablation*								

Hypo#	119.22 ± 23.99	80.14 ± 14.01	79.84 ± 18.80	22.87 ± 4.78	21.01 ± 4.42	18.36 ± 3.69	17.90 ± 4.64	39.08 ± 10.78
rhTSH##	121.17 ± 29.06	82.44 ± 17.84	80.49 ± 22.56	22.90 ± 5.29	21.47 ± 6.06	19.30 ± 4.17	18.78 ± 6.26	38.92 ± 12.19
p	0.749	0.526	0.892	0.980	0.703	0.292	0.481	0.953

Depression*								

No	130.25 ± 22.07	87.82 ± 13.36	87.00 ± 17.72	23.84 ± 4.49	23.57 ± 3.47	19.70 ± 3.75	20.72 ± 5.10	42.48 ± 9.88
Yes	108.16 ± 26.66	73.51 ± 15.55	71.97 ± 21.17	21.75 ± 5.42	18.46 ± 5.76	17.81 ± 3.97	15.50 ± 4.60	34.83 ± 11.92
p	**<0.001**	**<0.001**	**0.001**	0.065	**<0.001**	**0.033**	**<0.001**	**0.003**

Anxiety level**	-0.389	-0.408	-0.355	-0.250	-0.315	-0.326	-0.422	-0.326
p	**<0.001**	**<0.001**	**0.002**	**0.027**	**0.005**	**0.003**	**<0.001**	**0.004**

Interval surgery/^131^I**	0.005	0.010	-0.020	0.032	0.046	0.074	-0.098	-0.003
p	0.964	0.932	0.866	0.784	0.696	0.533	0.408	0.978

Baseline QOL**	0.574	0.618	0.531	0.385	0.601	0.483	0.630	0.473
p	**<0.001**	**<0.001**	**<0.001**	**<0.001**	**<0.001**	**<0.001**	**<0.001**	**<0.001**

#Hypo: ^131^I during hypothyroidism, ##rhTSH: ^131^I after rhTSH injections

PWB Physical well-being, SWB Social/family well-being, EWB Emotional well-being, FWB Functional well-being, FS Fatigue

FACIT-G (PWB, SWB, EWB, and FWB subscales), FACIT-F (FACT-G, and FS subscale), FACIT-F TOI (PWB, FWB, and FS subscales) the higher the score, the higher the QOL level

* mean ± standard deviation, p: p-value Student's t-test or Mann-Whitney test

** Pearson's correlation coefficient, p: p-value Pearson's test

Bold values: p < 0.05

**Table 3:** Factors linked to the 9-month FACIT scores/dimensions: multivariate analysis (standardized beta coefficient)

		FACIT-F	FACIT-G	FACIT-F TOI	PWB	SWB	EWB	FWB	FS
Gender (0 men, 1 women)	ß	-0.164	-0.128	-0.177	-0.230	0.050	-0.171	-0.097	-0.176

	p	0.112	0.187	0.098	**0.043**	0.572	0.125	0.321	0.111

Age group (0 <45 y, 1 ≥ 45 y)	ß	-0.223	-0.197	-0.236	-0.179	-0.163	-0.090	-0.202	-0.248

	p	**0.038**	**0.050**	**0.033**	0.119	0.076	0.413	**0.043**	**0.031**

Marital status (0 single, 1 partner)	ß	0.051	0.021	0.047	-0.010	0.017	0.059	0.040	0.089

	p	0.636	0.839	0.678	0.932	0.857	0.607	0.692	0.447

Depression (0 no, 1 yes)	ß	-0.026	-0.056	0.036	0.116	-0.441	0.199	-0.118	0.003

	p	0.838	0.698	0.818	0.471	**0.001**	0.213	0.409	0.984

Initial therapy (0 hypo, 1 rhTSH)	ß	-0.056	-0.029	-0.064	-0.041	-0.010	0.001	0.004	-0.070

	p	0.595	0.767	0.555	0.715	0.908	0.997	0.970	0.532

Anxiety level*	ß	-0.076	-0.061	-0.079	-0.165	0.128	-0.172	-0.076	-0.076

	p	0.588	0.649	0.587	0.284	0.285	0.258	0.565	0.616

Baseline QOL**	ß	0.444	0.499	0.442	0.380	0.513	0.500	0.447	0.357

	p	**0.002**	**<0.001**	**0.003**	**0.004**	**<0.001**	**0.001**	**<0.001**	**0.012**

PWB Physical well-being, SWB Social/family well-being, EWB Emotional well-being, FWB Functional well-being, FS Fatigue

FACIT-G (PWB, SWB, EWB, and FWB subscales), FACIT-F (FACT-G, and FS subscale), FACIT-F TOI (PWB, FWB, and FS subscales)

ß: standardized beta coefficient (ß represents the change in standard deviation units in QOL score resulting from a change of one standard deviation in the different independent variables);

p: p-value

*the higher the score, the higher anxiety level

**the higher the score, the higher the QOL level

Bold values: p < 0.05

## Discussion

In recent years, attention has been paid to the effect of treatment on QOL in cancer patients [25].

We have previously found as Pacini et al, that the use of rhTSH preserves the QOL of patients in the peri-ablation period [7,8]. To our knowledge, this is the first longitudinal study which assesses the determinant factors of QOL at the first post-ablation follow-up control and seems show that QOL at the first post-ablation follow-up (the overall 9-month QOL scores: FACIT-F, FACT-G, FACIT-F TOI) is not affected by modality of TSH stimulation prior to therapy and influenced only by patient age and the baseline QOL. Due to the lack of evaluation of baseline status, other QOL studies have not accounted for the potential impact of baseline QOL. In the present study, numerous confounding factors have been incorporated including socio-demographic parameters (age, gender, educational level, marital status, having children, occupational status), initial clinical characteristics (tumour staging, RAA therapy, surgery/^131^I time), and psychological variables (anxiety, depression) that aid in a more reliable assessment. Age at initial treatment is often quoted as a QOL predictive factor with older patients more vulnerable than the young [9,12-14]. As in other studies, gender and marital status [12,13] and educational level did not influence QOL [13]. In our study tumour stage and the thyroidectomy dynamic (one- or two-stage) also did not influence QOL outcome. However this conclusion should be qualified by the fact that our study cohort consisted of low-risk patients given that only one patient had persistent disease at 9 months and the two other patients with persistent metastatic lymph nodes underwent surgery during the first 6 months following ablation and were excluded from the analysis. Our results failed to demonstrate any influence of depression and anxiety on median-term QOL, domains often defined as independent parameters linked to QOL [13]. These last two factors may be of significant clinical value for health care workers. Despite these interesting findings, our study suffers from several limitations. The sample size was larger than the referenced prospective study [7] but too small to be compared to some cross-sectional studies [12,13]. In our multivariate analysis, several determinants might have been missed by the low statistical power and other studies with larger populations will be necessary. But this nevertheless represents an improvement on studies that do not take into account the potential QOL confounding factors [7,10,16]. The representativeness of our cohort may be questioned since it differs from the Pacini study which whilst using the same design comprised more male patients (20% versus 14.5%), and a lower mean age (43 versus 47 years). The comparison with cross-sectional studies is difficult because parameters have been collected at the time of the study and not at the time of the initial treatment resulting in a slightly older cohort than in our study [10,12,16]. Also our proportion of pT1 was higher than in Pacini's study, and we did not include pT1 stage and/or M1 disease. It is clear that there are multiple strategies for assessing QOL, using specific or generic questionnaires but it seems appropriate to adopt a cancer-specific instrument which is more sensitive for detecting and quantifying small changes [26]. Patients with low baseline QOL scores should be specificaly offered additional clinical support: cancer support groups for patients and families, more targeted cancer-related patient information, nurse and pyschologist's aides, participation in treatment decision-making.

## Conclusions

Medium- term QOL outcomes in thyroid cancer patients are mainly determined by pre-ablation QOL and seem unaffected by the modality of stimulation adopted. The assessment of baseline QOL may be a useful tool in order to target patients at risk of poor subjective outcomes.

## Consent statement

Written informed consent was obtained from the patient for publication of this case report and accompanying images. A copy of the written consent is available for review by the Editor-in-Chief of this journal.

## Competing interests

The authors declare that they have no competing interests.

## Authors' contributions

DT contributed to the study design, data collection, statistical analysis, interpretation of data and draft of the paper.

DT, KBB, CF, PA contributed to the study design, interpretation of data, draft of the paper and revision of the manuscript.

FS, JFH, CDM, OM contributed to the study design and patient's recruitment

FFP contributed to data analysis and interpretation of data.

All authors read and approved the final manuscript.
